# Impact of ginger powder (Zingiber officinale) supplementation on the performance, biochemical parameters, antioxidant status, and rumen fermentation in Ossimi rams

**DOI:** 10.14202/vetworld.2024.1619-1628

**Published:** 2024-07-26

**Authors:** Montaser Elsayed Ali, Sulaiman A. Alsalamah, Sarah A. Al-Thubyani, Narjes Baazaoui, Ahmed Ezzat Ahmed, Mohamed-Emad A. Nasser, Heba A. Nasr

**Affiliations:** 1 Department of Animal Productions, Faculty of Agriculture, Al-Azhar University, Assiut 71524, Egypt; 2 Department of Biology, College of Science, Imam Mohamad Ibn Saud Islamic University, Riyadh 11623, Saudi Arabia; 3 Department of Biology, College of Science, Taibah University, Madinah, Saudi Arabia; 4 Department of Biology, Applied College Muhayil Assir, King Khalid University, Abha 61421, Saudi Arabia; 5 Department of Biology, College of Science, King Khalid University, P.O. Box 9004, 61413 Abha, Saudi Arabia; 6 Department of Animal Productions, Faculty of Agriculture, Alexandria University, Egypt; 7 Department of Animal Medicine (Clinical and Laboratory Diagnosis), Faculty of Veterinary Medicine, Assiut University, 71526 Assiut, Egypt

**Keywords:** antioxidant status, biochemical parameters, ginger powder, rams, rumen fermentation

## Abstract

**Background and Aim:**

Ginger (*Zingiber officinale*) has great potential as a growth promoter and immunostimulant in ruminant nutrition. This study assessed the impact of ginger powder supplementation on Ossimi rams’ rumen fermentation, biochemical parameters, and antioxidant levels.

**Materials and Methods:**

Fifteen Ossimi rams, aged 10 ± 1.3 months and weighing 30 ± 1.5 kg. Rams were randomly divided into three experimental groups: The control group (G1) received standard feed, while ginger powder (5 g and 7 g/kg body weight [BW] for G2 and G3, respectively) mixed in water was administered to groups G2 and G3 before their standard feed.

**Results:**

The control group recorded higher dry matter (DM) intake values (p < 0.05) than the ginger-treated groups. The ginger-treated groups showed superiority (p < 0.05) in weight gain and feed conversion compared to the control group. The digestion coefficients of DM, crude protein, and crude fiber were significantly (p < 0.05) increased by a high dose (7 g/Kg BW) of ginger supplementation, whereas organic matter, ether extract, and nitrogen-free extract digestibility remained unchanged. Compared to the control group, the rams given 5 g of ginger had significantly less (p < 0.05) total protein and globulin in their serum, but the rams given 7 g of ginger had significantly more (p < 0.05) of these proteins. In the ginger groups, these levels were significantly (p < 0.01) lower than those in the control group for serum creatinine, uric acid, urea, total lipids, triglycerides, total cholesterol, glucose, serum alanine aminotransferase, aspartate aminotransferase, alkaline phosphatase, and gamma-glutamyl transferase. Rams given ginger had significant growth hormone, insulin-like growth factor-1, total superoxide dismutase, GSH-Px, TAC, immunoglobulin (Ig) A, and IgG enhancement (p < 0.01), and a decrease (p < 0.01) in malondialdehyde concentration compared to the control group. Significant increases in total short-chain volatile fatty acids, acetic, propionic, and isovaleric acids (p < 0.05), and significant decreases in NH3N and protozoa (p < 0.01).

**Conclusion:**

Ginger powder (5 g and 7 g) can improve growth, immune responses, antioxidant status, and ruminal parameters in rams. Further study is needed to evaluate the effect of ginger on different types of animals (cow, buffalo, and goat) to develop new feed additives.

## Introduction

Sheep convert forage into meat and milk, making them indispensable sources of protein in the human diet. Sheep are the most common animal raised in Egypt for meat [1–3]. Boosting the health and productivity of these animals benefits the human population [1–3]. Researchers have begun integrating alternative natural materials, such as medical herbs, into animal feed instead of antibiotics to mitigate health risks for animals and humans, reduce the development of antibiotic-resistant bacteria, and eliminate potential harmful residues in dairy and meat products. This led to the ban of the use of antibiotics in animal feed in the European Union by the European Union’s Agricultural Ministry on the first of January 2006 [4].

Cinnamon, oregano, thyme, ginger, garlic, and other herbs/spices possess health benefits (stimulating appetite/digestion, inhibiting microbes, reducing inflammation, neutralizing free radicals, and boosting immunity) when incorporated as feed additives in animal nutrition [5]. In particular, Ginger (*Zingiber officinale*) is highly valued for its beneficial properties. Ginger is a member of the *Zingiberaceae* family, with the genus name Zingiber [6]. Ginger, rich in gingerols and shogaols, is a significant source of potassium, magnesium (Mg), copper, and manganese. Ginger, in small quantities, contains vitamins A, E, B, and C [7]. Numerous studies have shown ginger possesses antioxidant, anti-inflammatory, and antimicrobial properties [8]. In response to a request from the European Commission, the European Food Safety Authority (EFSA), Panel recommended a maximum addition of 20 mg/kg ginger to the complete feed for sheep and goats [9].

This study investigated the production performance, rumen fermentation, kidney function, liver function, and antioxidant status in Ossimi rams supplemented with two doses (5 g and 7 g) of ginger powder to determine the optimal dose.

## Materials and Methods

### Ethical approval

The care and use of animals were approved by the ethics committee of Assiut University, Faculty of Veterinary Medicine, and practiced in accordance with the laws and regulations of Egypt (Approval no. 06/2024/0183).

### Study period and location

This study was conducted from March to May 2023 at the Agricultural Farm of Al-Azhar University, Assiut, Egypt.

### Animals management

The present study included 15 Ossimi rams equal in size, aged 10 ± 1.3 months and weighing 30 ± 1.5 kg. During the study, the animals feed individually in digestion boxes, during which time urine, manure, food intake, and remains are collected. The number of digestion boxes on the sheep farm was limited, so we selected the sample size accordingly. Before and during the study, all animals were healthy and had normal clinical indications. Before the study began, each rams received vaccinations compared with the majority of the illnesses and deworming in accordance with Veterinary advice. Oxytetracycline LA (1 mL/10 kg) was administered to the animals as a prophylactic treatment against bacterial disease. They were also treated for ecto- and endoparasites using Ivermectin (1 mL/10 kg body weight [BW]; Sigma Co. USA).

All animals were housed in individual pens with individual feeding and watering facilities. All animals were maintained under natural photoperiods and ambient temperatures. Fresh water was available at all times. All animals received a daily ration based on the National Research Council [10] for sheep. The rations were developed and comprised of concentrate mixture and roughage (wheat straw). The physical and chemical compositions of the experimental rations are listed in Table-1.

**Table-1:** Apparent digestibility of experimental rations.

Parameter	Concentrate mixture (%)	Wheat straw (%)
Dry matter	92.5	92.5
Organic matter	93.3	83.6
Crude protein	14	2.6
Crude fiber	2.81	0.68
Ether extract	7.3	36.30
Ash	6.7	10.11
Nitrogen free extract	69.19	44.02

### Experimental design

Before starting the study, rams were acclimatized for 1 week and, after that, were assigned randomly into three experimental groups: The first group (control group, G1) fed on a traditional ration and the second (G2) and the third (G3) groups were given ginger powder obtained from a local market, dissolved in a sufficient amount of water; in the morning before feeding on the traditionally offered ration. The doses of ginger dry powder were 5 g and 7 g/kg BW for G2 and G3, respectively. The animals were fed twice daily at 8:00 a.m. and 5:00 p.m., and any residues were collected and weighed throughout the experimental period. Animals were weighed at the beginning and end of the experiment (After 60 days of the treatment), and feed intake was recorded throughout the experimental period.

### Apparent digestibility trial

All experimental animals were used in the digestibility trial (after 2 months of the experiment). The digestibility trial consisted of 7 days as the collection period. Animals were weighed on the 1^st^ day of the primary and last days of the collection period. Feces were collected daily, every 24 h, in plastic bags and weighed. A 5% sample of the total daily feces of each animal was taken as a sample, sprayed with a solution of 10% formaldehyde, and 10% H_2_SO_4,_ and stored in an airtight container for chemical analysis. The digestion coefficients of nutrients for the different experimental rations were calculated using direct methods.

### Chemical analysis

Ration ingredients were sampled, dried, ground, and analyzed for nutrients. The total amount of the daily fecal matter excreted per animal was collected daily, weighed, recorded, and mixed thoroughly throughout the collection period, and representative samples were taken from each animal, dried for 24 h at 60°C, pooled together, mixed, ground and stored until analysis. The proximate compositions (dry matter [DM], crude protein [CP], ether extract, crude fiber, ash, and nitrogen-free extract) of the experimental diets and feces were determined according to Association of Official Analytical Chemists (AOAC) [11].

### Sampling and analysis of blood

Fifteen blood samples (5 animals × 3 groups × 1 time; 5 mL each) were individually collected from the jugular vein at the end of the experiment in the morning (2 h before meal). The blood samples were centrifuged at 3000× *g* for 15 min, and the resulting serum samples were harvested and stored at −20°C until further analysis.

The metabolic energy profile included data on glucose, cholesterol, triglycerides (TG), high-density lipoprotein (HDL) cholesterol, low-density lipoprotein (LDL) cholesterol, and very-LDL (VLDL) cholesterol; the metabolic protein profile included the metabolites total protein (TP), albumin, uric acid, urea, and creatinine; the metabolic mineral profile included data on calcium (Ca), phosphorus (P), and Mg; and the metabolic enzymatic profile included the enzymes aspartate aminotransferase (AST), gamma-glutamyl transferase (GGT), alkaline phosphatase (ALP), Alanine transferase (ALT). The analyses were performed using commercial kits (Spectrum Diagnostics, Cairo, Egypt) according to the manufacturer’s instructions specific to each metabolite. Serum globulin levels were calculated mathematically by subtracting albumin values from the total serum protein values, and the albumin-to-globulin (A/G) ratio was calculated by dividing the albumin value by the globulin value. The VLDL and LDL values were obtained using the equations proposed [10] based on total serum cholesterol, HDL, and TG: VLDL = TG/5; LDL = TC-HDL–VLDL.

Serum growth hormone (GH) and insulin-like growth factor-1 (IGF-1) concentrations were determined using enzyme-linked immunosorbent (ELISA) kits from Antibodies (A79860; Antibodies.com, Missouri, USA) and Cusabio Co. (E-EL-S1275; Wuhan, P.R. China), respectively.

### Oxidative stress biomarkers

Serum total antioxidant capacity (TAC), superoxide dismutase (SOD), glutathione peroxidase (GSH-Px), and Malondialdehyde (MDA) activities were analyzed colorimetrically by STAT LAB SZSL60-SPECTRUM using, Bio-Dignostic kits (Bio-Dignostic Company, Egypt).

### Immunoglobulin (Ig)

The concentrations of IgA and IgG were determined using a sandwich ELISA detection kit for bovine IgA and IgG against standards, according to the manufacturer’s suggested protocol (CUSAbio Biotech Inc., Wuhan, China).

### Sampling and analysis of rumen liquor

Toward the end of the digestion trials, the rumen liquor samples (about 100 mL) were taken from the experimental animals through a rubber stomach tube in a dry clean cup and taken to the laboratory for examination. The rumen liquor samples were taken before morning feeding. Rumen samples were immediately analyzed for pH by a digital pH-meter (Mettler-Toledo Ltd., England), and then samples were sieved through four-folds of sterile gauze and used as 2 mL fixed with strong acids to determine the volatile fatty acid concentration, 2 mL for determination of ammonia concentration, and 2 mL fixed and stained with methylene green formal saline for microscopic examination. Total rumen protozoal count according to Clergue *et al*. [11], biochemical examination including total volatile fatty acid (TVFAs) concentration estimated by Macro Kjeldahl steam distillation method as described by Hall *et al*. [12], and rumen ammonia nitrogen concentration estimated using kits produced by Spectrum Company, Egypt, according to the method of Zaki *et al*. [13].

### Economical evaluation

Economic gain was calculated as the market value of total income from total weight gain (TWG) after subtracting feed cost and the cost of medicinal plants (ginger dry powder) during the experimental period. Feed cost and cost per kg gain were calculated in Egyptian pounds.

### Statistical analysis

Data were subjected to analysis of variance using the Statistical Package for the Social Sciences version 20.0 (IBM Corp., NY, USA). Tukey’s range test was used for the mean value of each experimental group in the study. A p-value of 0.05 was considered significant.

## Results

### Growth performance

DM intake, average daily gain, and feed conversion of the different experimental groups are presented in Table-2. There were significant differences (p < 0.05) in DM intake between the different experimental groups and the groups treated with ginger had the lowest values compared to the control group. There were significant differences (p < 0.05) in the TWG between the experimental groups and the groups treated with ginger had the highest values compared with the control group. The groups treated with ginger showed significantly (p < 0.01) better feed conversion compared to the control group.

**Table-2:** Performance of rams supplemented by ginger powder.

Parameter	Groups			Standard error of the mean	p-value

	G1	G2	G3		
Initial body weight, kg	30	30.06	30	0.315	0.9761
Final body weight, kg	41.4^b^*	43.6^a^	43.8^a^	0.365	<0.0001
Total weight gain, kg	11.40^b^	13.54^a^	13.80^a^	0.515	0.0010
Concentration intake, g/h/d	681	715	705	15.051	0.1079
Wheat straw intake, g/h/d	340^a^	250^b^	260^b^	6.066	<0.0001
Dry matter intake, g/h/d Feed conversion	1021^a^ 5.44^a^	965^b^ 4.60^b^	965^b^ 4.21^b^	18.965 0.158	0.0172 <0.0001
Total feed intake cost, L.E	12.93	13.21	13.45		
Cost/kg gain, L.E	71.83	62.90	58.50		

^a,b^Means with different superscripts in the same row are significantly different (p < 0.05).

* Figures in the same row having the same superscripts are not significantly different

### Digestion coefficient of nutrients

The nutrient digestibility of rams supplemented with ginger is presented in Table-3. The digestion coefficient of nutrients was significantly (p < 0.05) affected by ginger supplementation and high-dose of ginger (7 g/kg BW) had higher values of the digestion coefficient of nutrients (DM, CP, and crude fiber [CF]), while no significant difference was observed in the organic matter (OM), ether extract (EE), and nitrogen free extract (NFE) digestibility.

**Table-3:** The digestion coefficient of nutrients of rams supplemented by ginger powder.

Parameter	Groups			Standard error of the mean	p-value

	G1	G2	G3		
Dry matter	52.73^b^*	58.74^ab^	62.13^a^	2.755	0.0375
Organic matter	59.03	61.10	65.31	4.068	0.3545
Crude protein	78.45^b^	82.36^a^	81.85^a^	0.799	0.0054
Ether extract	74.60	76.12	78.32	4.940	0.7605
Crude fiber	62.98^b^	67.51^ab^	69.69^a^	2.177	0.0540
Nitrogen free extract	56.21	59.15	64.33	2.759	0.0653

^a,b^Means with different superscripts in the same row are significantly different (p < 0.05).

* Figures in the same row having the same superscripts are not significantly different

#### Protein parameters

Serum protein constituents (Table-4) showed a significant decrease (p < 0.05) in total serum protein and globulin in the rams group supplemented with 5 g ginger, while a significant (p < 0.05) increase was observed in the rams groups supplemented with 7 g ginger and the control group. Serum albumin showed a significant (p < 0.05) increase in the ginger groups than in the control group. Regarding the serum A/G ratio, there was a significant (p < 0.05) difference between groups, and a high level was found in the group supplemented with 5 g ginger.

**Table-4:** Metabolic protein profile of rams supplemented by ginger powder.

Parameter	Groups			Standard error of the mean	p-value

	G1	G2	G3		
Total protein (g/dL)	8.04^a^	7.72^b^	8.23^a^	0.081	0.0024
Albumin (g/dL)	4.32^b^	4.73^a^	4.64^a^	0.057	0.0009
Globulin (g/dL)	3.72^a^	2.99^b^	3.59^a^	0.098	0.0007
Alb/Glb Ratio	1.16^b^	1.58^a^	1.30^b^	0.050	0.0004
Creatinine (mg/dL)	1.89^a^	1.79^a^	1.20^b^	0.079	0.0003
Uric acid (mg/dL)	3.40^a^	3.35^a^	3.2^b^	0.024	0.0005
Urea (mg/dL)	46.40^a^	45.30^b^	40.52^c^	0.052	<0.0001

^a,b,c^Means with different superscripts in the same row are significantly different (p < 0.05). *Figures in the same row having the same superscripts are not significantly different

### Kidney function

Serum creatinine, uric acid, and urea levels were significantly (p < 0.01) decreased in the ginger groups compared with the control group, especially with high doses (7 g/kg BW), as shown in Table-4. There were no significant differences in the creatinine and uric acid levels between the rams group supplemented with 5 g/kg BW and the control group.

### Metabolic energy

Rams supplemented with ginger (5 and 7 g/kg BW) showed a significant (p < 0.01) decrease in total lipids, TG, total cholesterol (TC), and glucose compared with the control group (Table-5). Furthermore, the ginger-treated groups showed a highly significant (p < 0.01) increase in HDL-cholesterol (HDL-C) and a high decrease (<0.01) in LDL-cholesterol and VLDL-cholesterol compared with the control group.

**Table-5:** Metabolic energy profile of rams supplemented by ginger powder.

Parameter	Groups			Standard error of the mean	p-value

	G1	G2	G3		
Total lipids (mg/dL)	41.53^a^*	32.71^b^	32.53^b^	0.258	<0.0001
Triglycerides (mg/dL)	26.83^a^	21.75^b^	20.98^b^	0.494	<0.0001
TC (mg/dL)	41.27^a^	36.83^c^	38.10^b^	0.351	<0.0001
HDL (mg/dL)	11.33^c^	12.28^b^	14.91^a^	0.138	<0.0001
LDL (mg/dL)	24.57^a^	20.19^b^	18.99^c^	0.127	<0.0001
VLDL (mg/dL)	5.37^a^	4.35^b^	4.20^b^	0.099	<0.0001
Glucose (mg/dL)	71.80^a^	65.13^b^	62.87^c^	0.221	<0.0001

TC=Total cholesterol, HDL=High density lipoprotein, LDL: Low density lipoprotein, VLDL: Very low density lipoprotein. ^a,b,c^Means with different superscripts in the same row are significantly different (p < 0.05).

* Figures in the same row having the same superscripts are not significantly different

### Liver function

The serum enzyme activities of rams supplemented with ginger are presented in Table-6. All treatment groups showed a significant (p < 0.01) decrease in serum alanine and ALT, AST, ALP, and GGT activities compared with the control group.

**Table-6:** Liver Functions and hormone activity of rams supplemented by ginger powder.

Parameter	Groups			Standard error of the mean	p-value

	G1	G2	G3		
ALT (IU/L)	50.42^a^*	49.59^b^	41.67^c^	0.219	<0.0001
AST (IU/L)	72.81^a^	71.51^b^	63.33^c^	0.189	<0.0001
ALP (IU/L)	7.12^a^	7.03^a^	5.60^b^	0.051	<0.0001
GGT (IU/L)	29.78^a^	27.76^b^	23.50^c^	0.161	<0.0001
GH (ng/mL)	5.50^b^	6.47^a^	6.32^a^	0.127	0.0006
IGF-1 (ng/mL)	57.55^c^	63.50^a^	61.87^ab^	0.303	<0.0001

ALT=Alanine aminotransferase, AST=Aspartate transaminase, ALP=Alkaline phosphatase, GGT=Glutamyl transferase, GH=Growth hormone, IGF-1=Insulin like growth factor-1. ^a,b,c^Means with different superscripts in the same row are significantly different (p<0.05).

* Figures in the same row having the same superscripts are not significantly different

### Hormone activity

There was a significant (p < 0.01) increase in the GH and IGF-1 in the ginger groups compared with the control one (Table-6).

### Immune activities

#### Antioxidants activity

The serum total SOD (T-SOD), glutathione peroxidase (GSH-Px), total antioxidant capacity (T-AOC), and MDA contents in rams supplemented with ginger powder are presented in Table-7. All treated groups showed a significant (p < 0.01) increase in T-SOD, GSH-Px, and TAC and a decrease (p < 0.01) in MDA concentration compared with the control group.

**Table-7:** Immune stimulatory effects of supplementation of ginger powder on rams.

Parameter	Groups			Standard error of the mean	p-value

	G1	G2	G3		
T-SOD (IU/mL)	10.11^c^*	14.48^b^	15.57^a^	0.094	<0.0001
GSH-Px (IU/mL)	150.00^c^	151.07^b^	152.20^a^	0.284	0.0008
TAC (IU/mL)	70.19^c^	72.27^b^	74.50^a^	0.272	<0.0001
MDA (nmol/mL)	4.47^a^	2.23^b^	2.17^b^	0.141	<0.0001
IgA (IU/L)	0.88^c^	1.09^b^	1.97^a^	0.028	<0.0001
IgG (IU/L)	1.08^b^	1.17^b^	2.35^a^	0.048	<0.0001

T-SOD=Total superoxide dismutase, GPx=Glutathione peroxidase, T-AOC=Total antioxidant capacity, MDA=Malondialdehyde, IgA=Serum immunoglobulin A, IgG=Serum immunoglobulin G, IgM=Serum immunoglobulin M. ^a,b,c^Means with different superscripts in the same row are significantly different (p < 0.05).

* Figures in the same row having the same superscripts are not significantly different

#### Ig

Concerning the serum immune response, ginger supplementation in rams significantly (p < 0.01) increased the cytokines levels of IgA and IgG compared with the control group (Table-7).

#### Blood minerals

The concentrations of Ca, P, and Mg in rams were significantly (p < 0.05) increased in treatment groups by ginger, especially high dose (7 g/kg BW) compared with the control, as shown in Table-8.

**Table-8:** Serum minerals of rams supplemented by ginger powder.

Parameter	Groups			Standard error of the mean	p-value

	G1	G2	G3		
Calcium (mg/dL)	8.55^c^*	9.33^b^	10.53^a^	0.202	0.0002
Phosphorus (mg/dL)	6.95^b^	7.50^b^	8.15^a^	0.212	0.0038
Magnesium (mg/dL)	3.27^b^	4.85^a^	5.07^a^	0.099	<0.0001

^a,b,c^Means with different superscripts in the same row are significantly different (p < 0.05).

* Figures in the same row having the same superscripts are not significantly different

#### Ruminal parameters

The ruminal parameters of rams supplemented with ginger powder are presented in Table-9. There were no significant differences in the rumen pH. There was a significant (p < 0.05) increase in the total short-chain volatile fatty acid, acetic, propionic, and isovaleric acids, while no significant differences were observed in the butyric, valeric acids, and acetate: propionate (C2:C3) ratio between the rams groups. Furthermore, ginger powder supplementation significantly decreased NH_3_N and protozoa levels compared with the control group.

**Table-9:** Ruminal parameters of rams supplemented by ginger powder.

Parameter	Groups			Standard error of the mean	p-value

	G1	G2	G3		
pH	6.50	6.70	6.61	0.035	0.1702
TVFAs (mEq/100 mL)	5.417^b^*	5.473^ab^	5.667^a^	0.074	0.034
Acetic acid (mEq/100 mL)	4.350^b^	4.363^b^	4.503^a^	0.030	0.0042
Propionic acid (mEq/100 mL)	1.95^b^	1.98^b^	2.06^a^	0.025	0.0122
Acetic/Propionic acid	2.233	2.207	2.187	0.024	0.2195
Butyric acid (mEq/100 mL)	1.00	1.003	1.037	0.019	0.1752
Valeric acid (mEq/100 mL)	0.261	0.263	0.273	0.013	0.6174
Isovaleric acid (mEq/100 mL)	0.123^b^	0.127^ab^	0.132^a^	0.002	0.0154
NH_3_N (mg/100 mL)	21.140^a^	20.403^b^	16.967^c^	0.644	0.0001
Protozoa (×10^5^ cell/mL)	4.737^a^	4.140^b^	3.957^c^	0.119	0.0001

^a,b,c^Means with different superscripts in the same row are significantly different (p < 0.05).

* Figures in the same row having the same superscripts are not significantly different

## Discussion

### Growth performance

In the ginger-supplemented groups, DM intake was significantly lower (p < 0.05) than in the other experimental groups. On the contrary, there were no significant differences in the intake of rams fed rations supplemented with different dosages of ginger [14]. Shams Al-Dain and Jarjeis [15] reported a notable enhancement of cows’ daily intake when provided with rations containing high and low amounts of ginger.

In comparison to the control group, groups supplemented with ginger had significantly greater (p < 0.05) TWG. Abo Bakr [14] found that the addition of ginger products in the supplementation enhanced rams growth performance. Shams Al-Dain *et al*. [16] reported significant (p ≤ 0.05) increases in average daily gain, total gain, and final weight of rams fed with 15 and 30 g of ginger/kg compared to the control group, consistent with our findings. The study findings of Oleforuh-Okoleh *et al*. [17] on broiler growth promotion using ginger and garlic were supported by the significant boost in daily weight gain and final BW in this study.

The significantly (p < 0.05) higher final weight and weight gain of rams supplemented with ginger can be attributed to their bioactive components, such as gingerol and shogaol. Gingerols and shagaols, the most powerful phenolic components in ginger, cause health benefits in both animals and humans [18, 19]. Medicinal plants can enhance digestive enzyme activity [20], thus boosting feed efficiency and growth performance [21]. Ginger rhizomes, rich in protease, improve protein digestion, enhance nutrient absorption from amino acids, and promote beneficial bacteria in the intestine [22]. The control group had poorer feed conversion than the ginger-supplemented groups (p < 0.01). Studies have shown that herbs used as animal feed additives can enhance feed conversion ratio, BW, feed intake, feed palatability, and animal acceptability [23].

### Digestion coefficient of nutrients

Consuming 7 g/kg BW of ginger led to increased nutrient (DM, CP, and CF) digestibility without affecting OM, EE, and NFE digestibility. Ginger, functioning as a rumen modifier, may contribute to enhanced digestibility of DM and fiber in herb-supplemented animals [24]. Ginger reportedly improves digestion and boosts DM and fiber digestibility [25]. Ginger, as an antioxidant, can boost the efficiency of the rumen by mitigating the harmful effects of excessive unsaturated fatty acids, as per Windisch *et al*. [26], and enhances pancreatic enzyme activity to improve digestion. Due to its phenolic nature and potency in stimulating feed digestion bacteria, Eugenol enhances protein digestibility [27].

### Biochemical parameters

#### Protein parameters

The findings corroborated earlier reports [28], indicating statistically significant enhancements in TPs and globulins subsequent to ginger powder administration (p < 0.05). The increase in TPs might be due to enhanced secretion of saliva, efficiency of digestion enzymes, digestion and metabolism, and slow time of feed passage, which increased the absorption of protein in the small intestine by ginger supplementation [25]. Blood albumin levels rose, possibly because of enhanced ruminal microbial protein synthesis, causing increased absorption [29].

Rams receiving high-dose ginger showed significantly (p < 0.05) higher globulin levels than the other treated groups. This increase can be attributed to ginger powder’s anti-inflammatory and immune-boosting properties in the body, which in turn increases globulin levels [30]. This study observed a statistically significant increase (p < 0.05) in serum A/G ratio with low-dose ginger intake. Compared to the control, a significant decline (p < 0.05) in the A/G ratio occurred following ginger supplementation [31].

## Kidney functions

Ginger’s polyphenols and flavonoids decrease serum creatinine, uric acid, and urea levels by influencing blood waste product removal. Ajith *et al*. [32] reported that ginger extract’s antioxidant nephroprotective effects and reductions of serum urea and creatinine levels could be attributed to the presence of polyphenols and flavonoids. Ginger’s inhibitory effect on ruminal deamination could account for the observed decrease in urea concentration [33]. With ginger oil supplementation, the serum urea level dropped significantly [14]. In the ginger groups, creatinine levels decreased compared to the control. Abo Bakr [14] found no significant difference in creatinine levels for ewes given water with ginger extract versus the control, but Nassar [33] reported a higher creatinine value in the ginger group.

The data obtained in this study regarding the effect of ginger on reduced uric acid levels confirmed the use of ginger as a therapeutic herb to remove uric acid from the body [34]. In rats and broilers, ginger and ginger oil lower uric acid levels [35]. Previously researched ginger components, including gingerol, shogaol, paradol, zingerone, flavonoids, polyphenols, riboflavin, and vitamins, have the ability to decrease uric acid levels [36].

### Metabolic energy profile

Serum triglyceride and cholesterol levels indicate the lipid metabolism status of animals. Reducing serum TG with ginger extract is linked to a decreased risk of metabolic diseases [37]. In accordance with Bhandari and Pillai [38], who proved the extract lessened serum TC and TG and amplified HDL-C levels. The observed reduction in triglyceride levels in rations supplemented with ginger may be due to ginger’s effects on liver tissues, its benefits in metabolism, and the negative effect of ginger on rumen microflora activity and digestion [14]. According to a prior investigation, ingestion of ginger daily reduced LDL, TC, and TG in serum while boosting HDL-C levels [39]. In addition, it was concluded that ginger’s hypocholesterolemic effect could result from inhibiting cellular cholesterol biosynthesis, increasing bile acid biosynthesis to eliminate cholesterol from the body, and increasing fecal cholesterol excretion after the consumption of ginger [40].

According to Farhan [41], Awassi lambs fed a ration with 5% or 10% ginger root had significantly lower blood glucose levels than reported, which agrees with our study. Recent studies indicate that ginger’s hypoglycemic effect may be attributed to its tannin and polyphenol content with antioxidant properties [42]. β-Sesquiphellandrene in ginger increases insulin sensitivity and contributes to its antidiabetic effect by inhibiting glucosidase [43]. The improvement in insulin sensitivity lowered circulating insulin levels as the cells no longer require as much insulin to be signaled [44]. In addition, the mechanism for reducing blood glucose by ginger through inhibition of hepatic phosphorylase enzyme, thereby preventing the breakdown of hepatic glycogen storages, increases the activity of enzymes, improving glycogen synthesis and suppressing the activity of hepatic “glucose 6-phosphatase” enzyme, that causes degradation of glucose 6-phosphate to glucose and, consequently increases blood glucose levels [45].

### Liver functions

Consuming ginger improved liver function, as indicated by decreased liver enzyme activity. Our findings agree with those of Novakovic *et al*. [46], showing decreased AST, ALT, ALP, and lipid peroxide levels after ginger administration. Another study found that ginger supplementation lowered AST, ALT, TG, and TC levels [46, 47]. While previous research by Fasseas *et al*. [31] and Zaki *et al*. [48] found no significant differences in AST, ALT, and GGT levels between ginger and control groups, contrastingly, Abo Bakr [14] observed notable rises.

### Hormone activity

Dietary ginger may improve nutrient usage and increase GH expression, with subsequent increases in IGF-1 and IGF-2 expression, indicating a physiological mechanism for better growth performance [49]. In this study, a larger body size was observed. The study by Yang *et al*. [50] revealed that enhancing serum IGF-1 concentrations in laying hens through herbal active ingredients led to improved production performance. In mammals, IGF1 regulates growth before and after birth [51]. It is the key mediator of GH function, overseeing tissue repair, intermediary metabolism, and disease pathogenesis throughout life [52]. An increase in IGF-1 expression can improve liver function and fibrosis, as demonstrated by Adamek and Kasprzak [53]. Reduced liver enzymes were observed in this study. IGF-1 enhances intestinal ion transport, boosting Ca absorption in animals [54]. In our study, there was a higher increase in serum Ca levels for treatment groups with ginger, especially at high doses, compared to the control.

### Immune activities

#### Antioxidants activity

These enzyme systems (SODs, GSH-PX, among others) in the body are induced by the presence of elevated free radical numbers to produce more antioxidants. This system’s augmented enzymatic activity signifies improved cellular defense against free radicals. According to reports, ginger reduces lipid peroxidation [8]. The strong antioxidant properties of ginger can be attributed to the presence of gingerol, as ginger exhibits various bioactivities, such as anti-inflammatory, antioxidant, growth promoter, and antimicrobial effects. The antioxidant properties of *Z. officinale* have been established through previous research by Heeba and Abd-Elghany [40]. Ginger promotes antioxidant defenses by stimulating antioxidant enzyme expression and decreasing reactive oxygen species generation and lipid peroxidation [55]. Ginger significantly enhanced antioxidant enzyme activities and levels of GSH and SOD [56]. Ginger has antioxidant activities due to the presence of tocopherols, phenols, and flavonoids; therefore, SOD activities may be mediated by these components present in the ginger extract [57].

#### Igs

The detection of IgA, IgG, and IgM in serum can represent the level of Ig in serum. The results of the study agreed with those reported by Nassar [33], who found that lambs fed ginger, either powder or oil additives, had higher IgG and IgG levels than the control group. In the feed mix, the addition of herbs led to an increase in the levels of IgA, IgG, and IgM, enhancement of the phagocytic activity of macrophages, and an increase in the number of stimulated B and T lymphocytes [58]. Animals fed ginger rations had higher IgG levels than the control group, which might be attributed to the effect of medicinal plants, which improve immunity [59].

#### Blood minerals

For ruminants, ginger consumption has led to enhanced serum mineral levels [60]. According to El-Gohary *et al*. [28], there was a significant increase (p < 0.05) of Ca in the blood of does, whereas ginger had no effect, according to Fasseas *et al*. [31], administering ginger to dairy cows did not alter their blood minerals. In the study by Afele *et al*. [56], serum Ca concentrations were significantly lower (p < 0.05) for mixed-breed rams in the ginger group. Previous studies by Al-Dain and Jarjeis [15] and Fasseas *et al*. [31] have revealed no impact of ginger supplement intake on Ca, P, and Mg levels.

#### Ruminal parameters

The response of the rumen to internal stability depends on ruminal pH, which should be maintained between 6.56 and 6.95. Previous studies by Fasseas *et al*. [31] and Zhang *et al*. [45] showed that ginger did not alter rumen pH. According to Fasseas *et al*. [31], the results of total short-chain TVFA in our study exhibited a slight increase, consistent with their findings. The levels of TVFA, acetate, propionate, and isovalerate found in this study correspond with Ikyume *et al*.’s [24] results, indicating enhanced microbial activity and dietary fermentability due to ginger consumption.

The decreased ammonia-N concentrations in the treated groups in this study could be attributed to the inhibition of protein hydrolyzing microorganisms in the rumen, as reported by Patra [61]. Ammonia nitrogen was affected by both ginger powder and oil additives and significantly decreased in groups fed ginger compared with the control group [33].

The addition of ginger powder to sheep diets resulted in a decrease in the TVFA concentration but no change in the acetate-to-propionate ratio, pH, or ammonia-N concentration [45]. With regard to Protozoa number, the reduction effect of ginger was clearly observed, and the number of protozoa was significantly decreased in the group supplemented with ginger compared with the control group. Ginger powder likely reduces microbial activity and diet fermentability because ginger increases feed stability and benefits the gastrointestinal ecology by inhibiting pathogenic microorganism growth [25]. The findings confirmed these observations, as the levels of protozoa in the rumen decreased, and the reduction rate increased with increasing ginger concentration. Previous studies by Muhammad *et al*. [60] and Soroor and Moeini [62] have reported that supplementation with ginger extracts decreases the protozoa population. The decreased number of total protozoa was probably due to secondary metabolites and antiprotozoal activities of ginger components [61]. Some previous studies by Fasseas *et al*. [31] found no significant difference (p > 0.05) in the total protozoal count in the rumen of sheep, whereas Patra *et al*. [61] demonstrated an increase in the protozoa count following the addition of ginger extract.

#### Economical evaluation

Feeding ginger to rams led to a higher total feed cost compared to the control group. The control group’s total feed cost per unit weight gain was greater than that of the treatment groups. According to Allam and El-Elaime [63], using certain medicinal herbs in rams rations enhanced the economic and relative efficiency of rams production.

## Conclusion

The present study revealed that ginger powder significantly improved ram growth, nutrient digestion efficiency, immune responses, antioxidant status, and ruminal fermentation. This study concludes that 5 or 7g/kg BW of ginger powder should be supplemented to rams. Finally, more research is needed to evaluate ginger’s effect on different farm animals and develop new feed additive.

## Authors’ Contributions

MEA: Statistical analysis and drafted the manuscript. HAN and MAN: Study design, collected the data, and revised the manuscript. SAA, SAAl, and NB: Data analysis interpretation, critically reviewed, and revised the manuscript. AEA: Statistical analysis and editing of the manuscript. All authors have read, reviewed, and approved the final manuscript.

## References

[1] Schwarz S, Kehrenberg C, Walsh T.R (2001). Use of antimicrobial agents in veterinary medicine and food animal production. Int. J. Antimicrob. Agents.

[2] Hoffmann E.M, Muetzel S, Becker K (2003). Effects of *Moringa oleifera* seed extract on rumen fermentation *in vitro*. Arch. Tierernahr.

[3] Hameed O, Hussain H, Al-Qudsei N.H (2012). Effect of adding ginger root in the concentrate diet on production of milk and its components in Holstein cattle. Al-Anbar J. Vet. Sci.

[4] Castanon J.I (2007). History of the use of antibiotic as growth promoters in European poultry feeds. Poult. Sci.

[5] El-Sayed S.M, Youssef A.M (2019). Potential application of herbs and spices and their effects in functional dairy products. Heliyon.

[6] Bakr A.F, Abdelgayed S.S, EL-Tawil O.S, Bakeer A.M (2020). Ginger extract and ginger nanoparticles;Characterization and applications. Int. J. Vet. Sci.

[7] Bhatt N, Waly M.I, Essa M.M, Ali A (2013). Ginger:A functional herb. In:Food as Medicine.

[8] Mao Q.Q, Xu X.Y, Cao S.Y, Gan R.Y, Corke H, Beta T, Li H.B (2019). Bioactive compounds and bioactivities of ginger (*Zingiber officinale* Roscoe). Foods.

[9] Bampidis V, Azimonti G, de Lourdes Bastos M, Christensen H, Kos Durjava M, Kouba M, López-Alonso M, Puente S.L, Marcon F, Mayo B, Pechová A, Petkova M, Ramos F, Sanz Y, Villa R.E, Woutersen R, Brantom P, Chesson A, Westendorf J, Gregoretti L, Manini P, Dusemund B (2020). Safety and efficacy of essential oil, oleoresin and tincture from *Zingiber officinale* Roscoe when used as sensory additives in feed for all animal species. EFSA J.

[10] Oliveira M.J, van Deventer H.E, Bachmann L.M, Warnick G.R, Nakajima K, Nakamura M, Sakurabayashi I, Kimberly M.M, Shamburek R.D, Korzun W.J, Myers G.L, Miller W.G, Remaley A.T (2013). Evaluation of four different equations for calculating LDL-C with eight different direct HDL-C assays. Clin. Chim. Acta.

[11] Clergue S.A.C, Depenbrock S.M, Chigerwe M (2023). Effect of sample volume and time on rumen juice analysis in cattle. J. Vet. Intern. Med.

[12] Hall M.B, Nennich T.D, Doane P.H, Brink G.E (2015). Total volatile fatty acid concentrations are unreliable estimators of treatment effects on ruminal fermentation *in vivo*. J. Dairy Sci.

[13] Zaki M, Baraka T, Tayeb F (2020). Acid-base balance, rumen, hematological, and serum biochemical parameters of healthy Egyptian Ossimi sheep. Vet. Med. J. Giza.

[14] Abo Bakr S (2019). Effect of adding ginger powder or ginger oil on productive performance of ewes during lactation period. Egypt. J. Nutr. Feeds.

[15] Al-Dain Q.Z.S, Jarjeis E.A (2015). Vital impact of using ginger roots powder as feed additive to the rations of local Friesian dairy cows and its effect on production and economic efficiency of milk and physiological of blood. Kufa J. Vet. Med. Sci.

[16] ShamsAl-Dain Q, Jarjeis E.A, Sulman H.A, Shailai M.H.A, Hamad Y.I (2018). Effect of adding ginger roots powder to the rations of Awassi lambs in some productive and physiological parameters under local condition of Nineveh province. Mesop. J. Agric.

[17] Oleforuh-Okoleh V.U, Chukwu G.C, Adeolu A.I (2014). Effect of ground ginger and garlic on the growth performance, carcass quality and economics of production of broiler chickens. Glob. J. Biosci. Biotechnol.

[18] Yu Y, Huang T, Yang B, Liu X, Duan G (2007). Development of gas chromatography-mass spectrometry with microwave distillation and simultaneous solid-phase microextraction for rapid determination of volatile constituents in ginger. J. Pharm. Biomed. Anal.

[19] Ding S.H, An K.J, Zhao C.P, Li Y, Guo Y.H, Wang Z.F (2012). Effect of drying methods on volatiles of Chinese ginger (*Zingiber officinale* Roscoe). Food Bioprod. Process.

[20] Mukherjee S, Mandal N, Dey A, Mondal B (2014). An approach towards optimization of the extraction of polyphenolic antioxidants from ginger (*Zingiber officinale*). J. Food Sci. Technol.

[21] Abedian Kenari A, Naderi M (2016). Effects of enriched *Artemia* by fish and soybean oils supplemented with vitamin E on growth performance, lipid peroxidation, lipase activity and fatty acid composition of Persian sturgeon (*Acipenser persicus*) larvae. Aquac. Nutr.

[22] Ali B.H, Blunden G, Tanira M.O, Nemmar A (2008). Some phytochemical, pharmacological and toxicological properties of ginger (*Zingiber officinale* Roscoe):A review of recent research. Food Chem. Toxicol.

[23] Valero M.V, do Prado R.M, Zawadzki F, Eiras C.E, Madrona G.S, do Prado I.N (2014). Propolis and essential oils additives in the diets improved animal performance and feed efficiency of bulls finished in feedlot. Acta Sci. Anim. Sci.

[24] Ikyume T.T, Afele T, Donkoh D.S, Aloko J.M, Suleiman U (2020). Comparative effect of garlic (*Allium sativum*) and ginger (*Zingiber officinale*) and their combination on growth, rumen ecology and apparent nutrient digestibility in sheep. Niger. J. Anim. Prod.

[25] Srinivasan K (2005). Spices as influencers of body metabolism:An overview of three decades of research. Food Res. Int.

[26] Windisch W, Schedle K, Plitzner C, Kroismayr A (2008). Use of phytogenic products as feed additives for swine and poultry. J. Anim. Sci.

[27] El-Essawy A.M, Abdou A.R, EL-Gendy M.H (2019). Impact of anise, clove, and thyme essential oils as feed supplements on the productive performance and digestion of Barki ewes. Aust. J. Basic Appl. Sci.

[28] El-Gohary E.S.H, EL-Saadany S.A, Abd-Elkhabeer M.A, Aiad K.M (2012). Effect of supplementing Som medicinal herbs and plants on the performance of lactating goats:1-productive and reproductive performance. J. Anim. Poult. Prod.

[29] Kholif S.M, Morsy T.A, Abdo M.M, Matloup O.H, Abu El-Ella A.A (2012). Effect of supplementing lactating goats rations with garlic, cinnamon or ginger oils on milk yield, milk composition and milk fatty acids profile. J. Life Sci.

[30] Fawzi E.M, Khalil A.A, Afifi A.F (2009). Antifungal effect of some plant extracts on *Alternaria alternat*a and *Fusarium oxysporum*. Afr. J. Biotechnol.

[31] Fasseas M.K, Mountzouris K.C, Tarantilis P.A, Polissiou M, Zervas G (2008). Antioxidant activity in meat treated with oregano and sage essential oils. Food Chem.

[32] Ajith T.A, Nivitha V, Usha S (2007). *Zingiber officinale* Roscoe alone and in combination with alpha-tocopherol protect the kidney against cisplatin-induced acute renal failure. Food Chem. Toxicol.

[33] Nassar M.S (2020). Adding Ginger powder or oil and its effect on nutritional evaluation of rams rations. Int. J. Environ. Agric. Biotechnol.

[34] Gehan A.E.E, Ayman Y.A (2010). Cadmium-ginger two way antagonistitc relationship. Arab. J. Biochem.

[35] Saeid J.M, Mohamed A.B, Al-Baddy M.A (2010). Effect of aqueous extract of ginger (*Zingiber officinale*) on blood biochemistry parameters of broiler. Int. J. Poult. Sci.

[36] Febriani Y, Riasari H, Winingsih W, Aulifa D.L, Permatasari A (2018). The potential use of red ginger (*Zingiber officinale* Roscoe) dregs as analgesic. Indones. J. Pharm. Sci. Technol.

[37] Xia Q, Lu F, Chen Y, Li J, Huang Z, Fang K, Hu M, Guo Y, Dong H, Xu L, Gong J (2024). 6-Gingerol regulates triglyceride and cholesterol biosynthesis to improve hepatic steatosis in MAFLD by activating the AMPK-SREBPs signaling pathway. Biomed. Pharmacother.

[38] Bhandari U, Kanojia R, Pillai K.K (2005). Effect of ethanolic extract of *Zingiber officinale* on dyslipidaemia in diabetic rats. J. Ethnopharmacol.

[39] Elshater A.E.A, Salman M.M.A, Moussa M.M.A (2009). Effect of ginger extract consumption on levels of blood glucose, lipid profile and kidney functions in alloxan induced-diabetic rats. Egypt. Acad. J. Biol. Sci. A Entomol.

[40] Heeba G.H, Abd-Elghany M.I (2010). Effect of combined administration of ginger (*Zingiber officinale* Roscoe) and atorvastatin on the liver of rats. Phytomedicine.

[41] Farhan A (2013). The vital effect of ground ginger roots on some physiological characteristics of the female Awassi lamb. Tikrit J. Agric. Sci.

[42] Ugwoke C.E.C, Nzekwe U (2010). Phytochemistry and proximate composition of ginger (*Zingiber officinale*). J. Pharm. Allied Sci.

[43] Hardianti S.C, Widiputri D.I, Gunawan M.D.P.T (2020). Process Development for The Production of *Cymbopogon Citratus* and *Zingiber Officinale* Roscoe Liquid Extracts in Herbal Industry.

[44] Sampath C, Rashid M.R, Sang S, Ahmedna M (2017). Specific bioactive compounds in ginger and apple alleviate hyperglycemia in mice with high fat diet-induced obesity via Nrf2 mediated pathway. Food Chem.

[45] Zhang T, Yang Z.B, Yang W.R, Jiang S.Z, Zhang G.G (2011). Effects of dose and adaptation time of ginger root (*Zingiber officinale*) on rumen fermentation. J. Anim. Feed Sci.

[46] Novakovic S, Jakovljevic V, Jovic N, Andric K, Milinkovic M, Anicic T, Pindovic B, Kareva E.N, Fisenko V.P, Dimitrijevic A, Joksimovic Jovic J (2024). Exploring the antioxidative effects of ginger and cinnamon:A Comprehensive review of evidence and molecular mechanisms involved in polycystic ovary syndrome (PCOS) and other oxidative stress-related disorders. Antioxidants (Basel).

[47] Ali M.E, Abd-Elkariem M.F.A, Fahmy S, Ali Hussein H, Abdelrahman M (2023). Dietary supplementation with different nano and organic selenium doses improves ovarian activity, fertility rate, and progesterone level in Ossimi ewes. Ital. J. Anim. Sci.

[48] Zaki M.G, Barka T.A, Tayeb F.A.E.F (2021). Effect of ginger powder (*Zingiber officinale*) on acid-base balance, rumen and blood constituents in healthy Egyptian sheep. Int. J. Vet. Sci.

[49] Midhun S.J, Arun D, Edatt L, Sruthi M.V, Thushara V.V, Oommen V.O, Sameer Kumar V.B, Divya L (2016). Modulation of digestive enzymes, GH, IGF-1 and IGF-2 genes in the teleost, Tilapia (*Oreochromis mossambicus*) by dietary curcumin. Aquac. Int.

[50] Yang J.X, Chaudhry M.T, Yao J.Y, Wang S.N, Zhou B, Wang M, Han C.Y, You Y, Li Y.E (2018). Effects of phyto-oestrogen quercetin on productive performance, hormones, reproductive organs and apoptotic genes in laying hens. J. Anim. Physiol. Anim. Nutr. (Berl).

[51] Kasprzak A, Kwasniewski W, Adamek A, Gozdzicka-Jozefiak A (2017). Insulin-like growth factor (IGF) axis in cancerogenesis. Mutat. Res. Rev. Mutat. Res.

[52] Durzyńska J (2014). IGF axis and other factors in HPV-related and HPV-unrelated carcinogenesis (Review). Oncol. Rep.

[53] Adamek A, Kasprzak A (2018). Insulin-like growth factor (IGF) system in liver diseases. Int. J. Mol. Sci.

[54] Sutter N.B, Bustamante C.D, Chase K, Gray M.M, Zhao K, Zhu L, Padhukasahasram B, Karlins E, Davis S, Jones P.G, Quignon P, Johnson G.S, Parker H.G, Fretwell N, Mosher D.S, Lawler D.F, Satyaraj E, Nordborg M, Lark K.G, Wayne R.K, Ostrander E.A (2007). A single IGF1 allele is a major determinant of small size in dogs. Science.

[55] Hussein H.A, Hassaneen A.S.A, Ali M.E, Sindi R.A, Ashour A.M, Fahmy S.M, Swelum A.A, Ahmed A.E (2022). The impact of rumen-protected L-arginine oral supplementation on libido, semen quality, reproductive organ biometry, and serum biochemical parameters of rams. Front. Vet. Sci.

[56] Afele T, Ikyume T.T, Allu R.P, Aniche O.S, Onuh M.E, Agbo E (2020). Effects of garlic (*Allium sativum*) and ginger (*Zingiber officinale*) powder and their combination on antioxidant and hematological response in sheep. Malays. J. Anim. Sci.

[57] Sukumaran V, Park S.C, Giri S.S (2016). Role of dietary ginger *Zingiber officinale* in improving growth performances and immune functions of *Labeo rohita* fingerlings. Fish Shellfish Immunol.

[58] Ozkaya S, Erbas S, Ozkan O, Baydar H, Aksu T (2017). Effect of supplementing milk replacer with aromatic oregano (*Oreganum onites* L.) water on performance, immunity and general health profiles of Holstein calves. Anim. Prod. Sci.

[59] Stef L, Dumitrescu G, Drinceanu D, Ştef D, Mot D, Julean C, Tatileanu R, Corcionivoschi N (2009). The effect of medicinal plants and plant extracted oils on broiler duodenum morphology and immunological profile. Rom. Biotechnol. Lett.

[60] Muhammad N, Ibrahim U.M, Maigandi S.A, Abubakar I.A (2016). Live performance and rumen microbial composition of Yankasa rams with supplemented levels of *Zingiber officinale*. J. Agric. Ecol. Res. Int.

[61] Patra A.K, Kamra D.N, Agarwal N (2010). Effects of extracts of spices on rumen methanogenesis, enzyme activities and fermentation of feeds *in vitro*. J. Sci. Food Agric.

[62] Soroor M.E.N, Moeini M.M (2014). The influence of ginger (*Zingiber Officinale*) on *in vitro* rumen fermentation patterns. Annu. Res. Rev. Biol.

[63] Allam S, El-Elaime R.R (2020). Impact of garlic, lemongrass, peepermint and rosemary as feed additives on performance of growing Barki lambs. Egypt. J. Nutr. Feeds.

